# Distal locking technique affects the rate of iatrogenic radial nerve palsy in intramedullary nailing of humeral shaft fractures

**DOI:** 10.1007/s00402-022-04665-1

**Published:** 2022-10-31

**Authors:** Fabian Greiner, Georg Kaiser, Anne Kleiner, Jonas Brugger, Silke Aldrian, Reinhard Windhager, Stefan Hajdu, Markus Schreiner

**Affiliations:** 1 Department of Orthopedics and Trauma Surgery, Medical University of Vienna, Währinger Gürtel 18-20, 1090 Vienna, Austria; 2 Center for Medical Statistics, Medical University of Vienna, Währinger Gürtel 18-20, 1090 Vienna, Austria

**Keywords:** Trauma, Humerus, Intramedullary nailing, Nerve injury, Fracture

## Abstract

**Background:**

Intramedullary humeral nailing is a common and reliable procedure for the treatment of humeral shaft fractures. Radial nerve palsy is a common complication encountered in the treatment of this pathology. The radial nerve runs from posterior to anterior at the lateral aspect of the distal humerus. Hence, there is reason to believe that due to the anatomic vicinity of the radial nerve in this area, lateral–medial distal locking in intramedullary nailing of the humerus may be associated with a greater risk for iatrogenic radial nerve injury compared to anterior–posterior locking.

**Questions/purpose:**

To assess whether the choice of distal locking (lateral–medial versus anterior–posterior distal locking) in intramedullary humeral nailing of humeral shaft fractures affects the risk for iatrogenic radial nerve injury.

**Patients and methods:**

Overall, 203 patients (116 females, mean age 64.3 ± 18.6 years), who underwent intramedullary nailing of the humerus between 2000 and 2020 at a single level-one trauma center, met the inclusion criteria and were analyzed in this retrospective case–control study. Patients were subdivided into two groups according to the distal locking technique.

**Results:**

Anterior–posterior locking was performed in 176 patients versus lateral–medial locking in 27 patients. We observed four patients with iatrogenic radial nerve palsy in both groups. Risk for iatrogenic radial nerve palsy was almost 7.5 times higher for lateral–medial locking (OR 7.48, *p* = 0.006). There was no statistically significant difference regarding intraoperative complications, union rates or revision surgeries between both groups.

**Conclusions:**

Lateral–medial distal locking in intramedullary nailing of the humerus may be associated with a greater risk for iatrogenic radial nerve palsy than anterior–posterior locking. Hence, we advocate for anterior–posterior locking.

**Level of evidence:**

Level III retrospective comparative study.

## Introduction

**Fig. 1:**
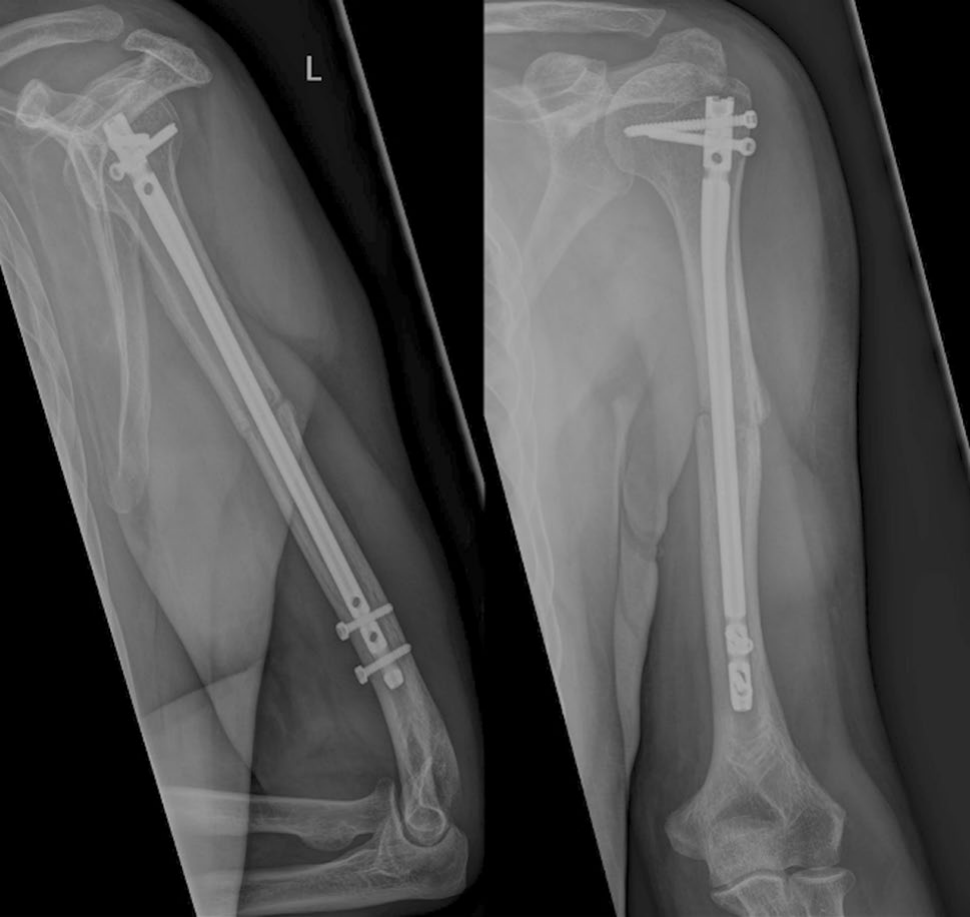
postoperative a.p. and lateral radiograph showing anterior–posterior locking technique

**Fig. 2:**
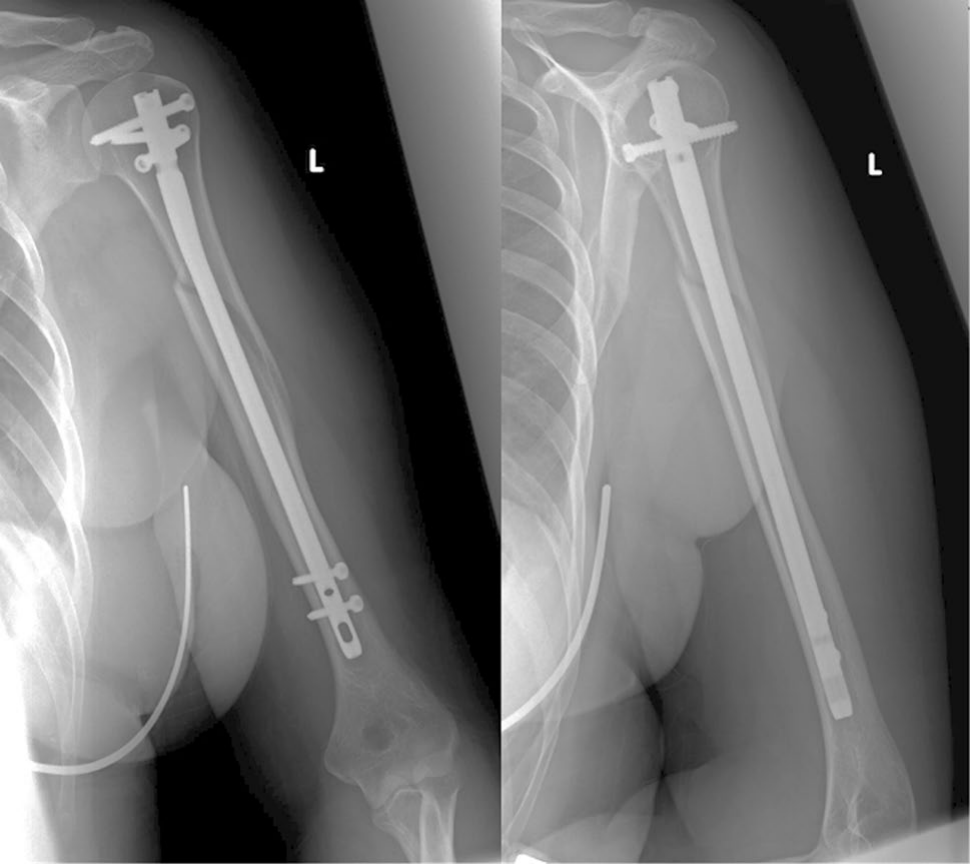
postoperative a.p. and lateral radiograph showing lateral–medial locking technique

Diaphyseal humeral fractures are a commonly seen injury in fracture clinics all around the globe. They account for 3–5% of all fractures in the human body. [9] The risk is bimodally distributed, with young men aged under 35 years and women over 50 years at an increased risk for humeral shaft fractures [5, 9, 16]. Because of the anatomic vicinity of the radial nerve to the humeral shaft, radial nerve palsies are the most common nerve injury associated with any fracture [8, 22, 27]. Incidence in literature varies between 2 and 17%. [10, 14, 20, 27] Humeral shaft fractures can be treated conservatively or operatively. Open reduction and internal fixation (ORIF) was the “gold-standard” of treatment for many years but requires surgical experience and meticulous care of anatomic structures [25]. Intramedullary nailing (IM Nailing) initially became more popular in the treatment of fractures of the femur and the tibia. Subsequently, it also became available for the treatment of humeral shaft fractures [7, 11] This technique met the need for a less invasive approach, thus reducing excessive soft tissue damage and fostered a reduction in common complications. The risk of iatrogenic radial nerve palsy (IRNP), however, remains a common and devastating problem. And, while a lower incidence for IRNP has been reported with IM nailing compared with open reduction and plate osteosynthesis [2], other sources have reported similar rates for both procedures [19, 26]. Previous studies described the incidence of this complication after surgical treatment at about 7% [4, 26, 28]. Many studies already tried to define safe zones in surgical approaches or pin placement for external fixation to avoid the radial nerve in the distal aspect of the humerus. Distal locking of intramedullary nails can either be performed in anterior–posterior or lateral-medial technique (see Figs. 1, 2).

The choice of locking is influenced by the implant, the fracture and the surgeon’s own preference. Although many studies already described techniques to avoid the radial nerve for pin placement in external fixation, there is no study to date that focuses on the risk for an IRNP for anterior–posterior and lateral–medial locking in intramedullary nailing [1, 15, 29, 30]. The radial nerve runs in the radial nerve sulcus from posterior to anterior in the distal third of the humerus [30]. On the basis of its anatomic course we hypothesized that due to the vicinity of the nerve in lateral–medial locking screw placement, the radial nerve is put at risk resulting in increased rates of surgery-associated radial nerve palsy compared to anterior–posterior locking.

Hence, the purpose of this present study was to assess whether the choice of distal locking (lateral–medial versus anterior–posterior) in intramedullary humeral nailing of humeral fractures affects the risk for iatrogenic radial nerve injury.

## Materials and methods

This study was approved by the local ethic committee and was performed in accordance with the Declaration of Helsinki for ethical principles for medical research involving human subjects. We retrospectively identified all patients with humeral shaft fractures who underwent intramedullary nail fixation during a 20-year period (01/01/2000–31/12/2020) at our level-one trauma center. Patients were identified using the hospital’s diagnostic and procedural codes. Exclusion criteria included primary radial nerve palsy, pathologic fractures, former pathologies or nerval injuries to the fractured extremity as well as polytraumatized patients. Datasets were then reviewed for completeness and accuracy. Patients with incomplete datasets were excluded. We then reviewed patient’s charts and admission x-rays for the fracture mechanism, the fracture classification according to the classification of the Arbeitsgruppe für Osteosynthesefragen (AO), the used implant and the duration of surgery. IM Nailing was done in either antegrade or retrograde technique. In antegrade nailing, the T2 Humeral Nailing System (Stryker Corporation, Kalamazoo, Michigan, US) was used. This implant allows for either a.p. or lateral locking (see Figs. 1, 2). Retrograde Nailing was performed with the unreamed Humeral Nail (UHN) (DePuy Synthes, Raynham, Massachusettes, US), which only allows for a.p. locking. All locking bolts either lateral–medial and anterior–posterior were placed through an incision and not percutaneously. All Patients’ primary examination, surgical and follow-up reports were reviewed for documentation of radial nerve palsy in physical examination. Radial nerve palsy was characterized as full loss of motoric function of the radial nerve. We reviewed surgical reports and postoperative x-rays to determine which kind of distal locking technique was used. Furthermore, intraoperative as well as postoperative complications including non-union and need for revision surgery were assessed for all patients.

### Statistics

After data entry, a cross-table analysis was performed to calculate the odds for the occurrence of a radial nerve palsy after anterior–posterior and lateral–medial locking to test our hypothesis.

Significance level for testing was set to *α* = 0.05. A logistic regression analysis was fitted to check for confounding variables and other risk factors (sex, trauma machanism, age, nailing technique, time to surgery, fracture pattern). Statistical analysis was performed using SPSS Statistics Version 27.0 (IBM, Armonk, New York, US).

### Patients

After the application of inclusion and exclusion criteria, 203 patients, who underwent intramedullary nailing for a humeral shaft fracture between 2000 and 2020 could be included in this study. For demographic data of the patient, collective see Table 1. Table 2 gives the flowchart of included patients.

**Table 1:** Demographic data of included patients

Patients	203
Male	87 (42.9%)
Female	116 (57.1%)
Age, years	64.08 ± 18.6 (range 19 to 97)
Injured side, left	106 (52.2%)
High energy Trauma	22 (10.8%)
Low energy Trauma	181 (89.2%)
Time to surgery, days	3.47 (range 0 to 28)

**Table 2:**
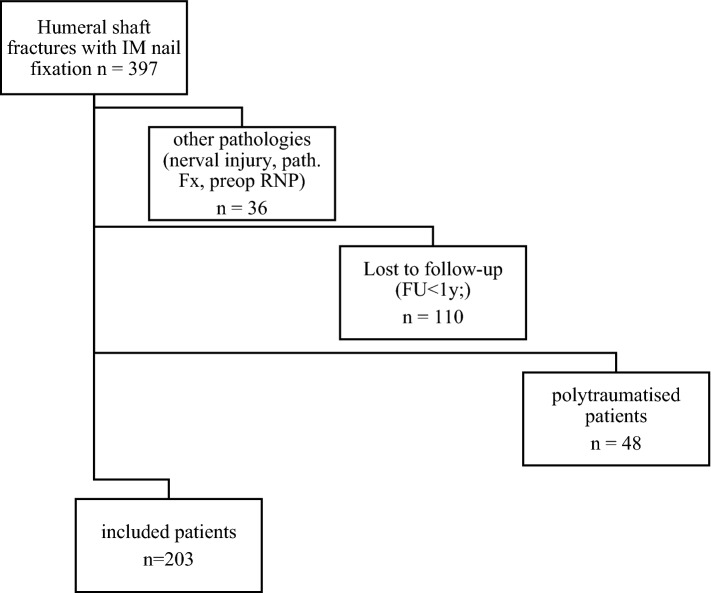
Flowchart of included patients

## Results

Most common fracture pattern was A1 (28.1%) and B1 (24.1%) according to the classification by the “Arbeitsgemeinschaft für Osteosynthesefragen” (Table 3). All fractures were closed fractures. Mean age of patients was 64.3 ± 18.6 years (range 19 to 97). There were 116 females and 87 males (57.1%/42.9%). Mean time to surgery was 3.47 days. Mean time to surgery was 105.3 ± 37.5 min.

**Table 3:** Fracture Patterns according to the AO Classification

	Total
AO classification	
A1	57 (28.1%)
A2	33 (16.3%)
A3	26 (12.8%)
B1	49 (24.1%)
B2	15 (7.4%)
B3	6 (3.0%)
C1	15 (7.4%)
C2	1 (0.5%)
C3	1 (0.5%)

### Iatrogenic radial nerve palsy after intramedullary nailing

In 203 patients we identified 8 patients with an iatrogenic radial nerve palsy. This makes for an absolute risk for the appearance of an IRNP after intramedullary nailing of 3.9%. Anterior–posterior locking was used more frequently than lateral–medial locking. 176 patients (86.7%) underwent anterior–posterior and 27 patients (13.3%) lateral–medial locking. Of 8 patients with IRNP, four patients had anterior–posterior locking and four patients lateral–medial locking. In Table 4 all patients with IRNP are listed individually. Risk to develop an IRNP was 2.3% for anterior–posterior and 14.8% for lateral–medial locking (Table 5). This makes for a 7.48 times higher risk to suffer a radial nerve palsy with lateral–medial locking compared to anterior–posterior locking (OR 7.48, *p* = 0.006). Two of eight patients underwent surgical revision after the primary operation to examine the radial nerve. In one patient the radial nerve was found to be intact, in the second patient the nerve was near the distal lateral locking screw and elongated showing signs of macroscopic injury. This patient was found to be the only of all eight patients who showed residual loss of radial nerve function at 1-year follow-up. Duration until full remission of symptoms took on average 12.9 weeks among the other seven patients (range 4 to 32 weeks).

**Table 4:** Observed radial nerve palsies in the two different locking technique

Patient	Sex	Age	AO class	Locking
1	W	58	B2	Anterior–posterior
2	W	68	B1	Lateral–medial
3	W	65	C1	Lateral–medial
4	M	73	A1	Lateral–medial
5	W	73	B1	Anterior–posterior
6	W	30	A2	Anterior–posterior
7	W	76	B1	Lateral–medial
8	M	74	A2	Anterior–posterior

**Table 5:** Individual patients with iatrogenic radial nerve palsy

Locking	Secondary RNP	Total
Anterior–posterior	4 (2.3%)	176 (86.7%)
Lateral–medial	4 (14.8%)	27 (13.3%)
Total	8 (3.9%)	203

### Further possible risk factors

We also included sex, trauma machanism, age, nailing technique, time to surgery and fracture pattern in the search for risk factors for the occurrence of iatrogenic radial nerve palsy. None of these factors could be identified as a risk factor by the conducted regression analysis. Females showed a higher risk of secondary radial nerve palsy (OR 2.20, *p* > 0.05), however not significant.

### Complications

Complications with the need for revision surgery were found in 12 patients with the leading cause being pseudarthrosis with the need for metal removal and plate osteosynthesis. No difference was found in the rate of complications between the two groups. The number of all complications is given in Table 6.

**Table 6:** Other complications

Complication	Number
Pseudarthrosis	5
Secondary dislocation	2
Intraoperative Fx	1
Hematoma	1
Active bleeding	1
Osteolysis	1
Periimplantary Fx after fall	1

### Metal removal

Of 203 patients 18 patients underwent a second surgery for full or at least partial metal removal. 12 patients underwent nail removal after fracture healing, six underwent removal of at least one locking screw due to screw loosening.

## Discussion

This study compared the risk for iatrogenic radial nerve palsy in anterior–posterior and lateral–medial distal locking for intramedullary nailing of humeral shaft fractures. The main finding of this study was the significantly higher risk for iatrogenic radial nerve palsy after lateral–medial versus anterior–posterior distal locking for intramedullary nailing of humeral shaft fractures. Overall risk for the development of radial nerve palsy after humeral nailing was 3.8% among patients. Similar rates of iatrogenic radial nerve palsy are reported in the literature. Studies, which assessed the risk for iatrogenic radial nerve palsy in patients undergoing operative fixation of humeral shaft fractures (ORIF and IM nailing) report numbers between 4.2 and 7% [4, 32]. Risk for iatrogenic radial nerve palsy after intramedullary nailing has been more recently reported to vary between 1 and 5% in other studies [2, 12, 18, 24]. A systematic review by Beeres et al. found an overall risk of 6.9% for plate fixation and 2.5% for intramedullary nailing in 26 included studies. [2]

In the present study, we observed a higher risk for the development of an iatrogenic radial nerve palsy after lateral to the medial insertion of the distal locking screws as compared to anterior–posterior insertion. Earlier anatomic studies already described that the radial nerve is endangered with lateral to medial distal locking [21]. To our knowledge, this is the first clinical study to describe and confirm this finding. Various cadaveric studies described the course of the radial nerve with respect to the humerus. It is most vulnerable at the transition from posterior to anterior on the lateral aspect of the humerus, where it pierces the intramuscular septum because it is tethered at this point and cannot avoid potential incoming foreign material (e.g., pins, screws) [6]. In an attempt to define and describe safe zones of pin placement for external fixation Theeuwes et al. dissected 20 arms and described this point of risk at an average height of 66 mm measured from the radial epicondyle, but this comes with great variation [30]. In 2014, Wegmann et al. published an anatomic study on 95 upper limbs showing the high variability of the radial nerve making it not possible to describe safe zones for pin placement [34]. An often-cited paper in this field from Kamineni et al. [15] back in 2009 described the transepicondylar distance, stating that there is a correlation between the distance of the radial and medial epicondyle and the point where the radial nerve runs from posterior to anterior. They allege that the caudad 70% of a longitudinal line equivalent in length and 90° onto the patient’s own transepicondylar distance are safe for screw insertion. Despite all attempts to identify safe zones, the main recommendation when using lateral locking screws or pin insertion is to make a larger skin incision and visualize the underlying soft tissue to minimize risk for radial nerve injury. Percutaneous minimal invasive techniques are not appropriate for lateral screw insertion and thus should not be used. At our department it is standard practice to use a single incision with visualization of the underlying soft tissue and direct bone contact with the drill sleeve to the humerus while drilling holes for lateral locking screws. Still we observed four patients with postoperative radial nerve palsy in lateral–medial locking. We hypothesize that this is due to the pull from retractors or the postoperative hematoma in the nerves proximity.

The proximal–distal position of the distal locking screws might also affect the risk for iatrogenic radial nerve palsy. And it is worth noting, that the distal locking holes for the antegrade humeral nail are arranged in alternating fashion, providing two holes for each technique with a marginal spacing of 2.5 mm between a.p. and lateral locking holes. The anterior–posterior locking holes of the retrograde humeral nail (DePuy Synthes, Raynham, Massachusettes, US) are within 3 mm of the anterior–posterior locking holes of the antegrade nail (distance measured from the respective nail tip). However, given the known variance of the course of the radial nerve, we do not think that this difference influences the rate of RNP.

Most radial nerve palsies remain transient. In our study cohort, only one patient showed residual signs of persisting radial nerve palsy at 1 year. Therefore, direct injury to the nerve whilst visualizing the humerus or damage during drilling seems unlikely in the remaining patients. The high rates of spontaneous nerve recovery are reported in earlier studies [13, 17, 27]. In accordance with existing literature, in our study seven out of eight IRNP resolved spontaneously after a maximum of 32 weeks. There is still a debate about when to explore the radial nerve. Most institutions advocate an conservative therapy scheme given the high number of spontaneous recoveries. Supporters of a primary exploration on the other hand mention that if the radial nerve is severely injured, primary exploration allows for early classification of the nerve injury itself without the need for a secondary exploration after missing recovery [3, 23]. Nevertheless, acute exploration after secondary radial nerve palsy is not recommended in current literature at this time [31, 33]. It must be pointed out that while lateral–medial locking endangers the radial nerve greatest care must also be taken when using anterior–posterior locking to not damage neurovascular structures. Structures at risk include the musculocutaneous and median nerve as well as the brachial artery. Direct visualization of the bone while drilling remains obligatory in both locking techniques to avoid these injuries.

This study must be interpreted in light of its limitations. The biggest limitation is its retrospective design. However, the greatest care was taken to include only patients with complete documentation and follow-up, which on the other hand entails a high number of excluded patients. Furthermore, the number of patients undergoing lateral to medial locking technique was low compared to anterior–posterior locking. Moreover, the number of observed radial nerve palsies likewise was relatively low. Therefore, the statistical analysis should be interpreted with caution and these low numbers all lead to a lack of power in this study which must be kept in mind when reading these results. In addition, patients were treated over two decades by different surgeons with two different nailing techniques which generate the risk of further confounders.

## Conclusion

To our knowledge, this is the first study to report a higher risk of iatrogenic radial nerve palsy caused by lateral–medial versus anterior–posterior distal locking in intramedullary nailing. This is most likely explained due to the anatomic course of the radial nerve and its immediate proximity to the location where lateral locking screws are usually inserted. Hence, we advocate for anterior–posterior distal locking in intramedullary humeral nailing. Still greatest care must be taken to avoid neurovascular injury in both distal locking techniques.
